# Zinc Intake and Risk of Prostate Cancer: Case-Control Study and Meta-Analysis

**DOI:** 10.1371/journal.pone.0165956

**Published:** 2016-11-08

**Authors:** Abeer M. Mahmoud, Umaima Al-Alem, Firas Dabbous, Mohamed M. Ali, Ken Batai, Ebony Shah, Rick A. Kittles

**Affiliations:** 1 Department of Kinesiology and Nutrition and Department of Physical Therapy, School of Applied Health Sciences, University of Illinois at Chicago, Chicago, Illinois, United States of America; 2 Department of Pathology, South Egypt Cancer Institute, Assiut University, Assiut, Egypt; 3 Division of Epidemiology and Biostatistics, School of Public Health, University of Illinois at Chicago, Chicago, Illinois, United States of America; 4 James R. & Helen D. Russell Institute for Research & Innovation, Advocate Lutheran General Hospital, Park Ridge, Illinois, United States of America; 5 Department of Surgery, College of Medicine, University of Arizona, Tucson, Arizona, United States of America; University of South Alabama Mitchell Cancer Institute, UNITED STATES

## Abstract

Zinc is an essential dietary element that has been implicated in the pathogenesis of prostate cancer, a cancer that disproportionately affects men of African descent. Studies assessing the association of zinc intake and prostate cancer have yielded inconsistent results. Furthermore, very little is known about the relationship between zinc intake and prostate cancer among African Americans. We examined the association between self-reported zinc intake and prostate cancer in a hospital-based case-control study of African Americans. We then compared our results with previous studies by performing a meta-analysis to summarize the evidence regarding the association between zinc and prostate cancer. Newly diagnosed African American men with histologically confirmed prostate cancer (n = 127) and controls (n = 81) were recruited from an urban academic urology clinic in Washington, DC. Controls had higher zinc intake, with a mean of 14 mg/day versus 11 mg/day for cases. We observed a non-significant, non-linear increase in prostate cancer when comparing tertiles of zinc intake (OR _<6.5 vs 6.5–12.5mg/day_ 1.8, 95% CI: 0.6,5.6; OR _<6.5 vs >12.5mg/day_ 1.3, 95% CI: 0.2,6.5). The pooled estimate from 17 studies (including 3 cohorts, 2 nested case-control, 11 case-control studies, and 1 randomized clinical trial, with a total of 111,199 participants and 11,689 cases of prostate cancer) was 1.07_hi vs lo_ 95% CI: 0.98–1.16. Using a dose-response meta-analysis, we observed a non-linear trend in the relationship between zinc intake and prostate cancer (p for nonlinearity = 0.0022). This is the first study to examine the relationship between zinc intake in black men and risk of prostate cancer and systematically evaluate available epidemiologic evidence about the magnitude of the relationship between zinc intake and prostate cancer. Despite of the lower intake of zinc by prostate cancer patients, our meta-analysis indicated that there is no evidence for an association between zinc intake and prostate cancer.

## Introduction

Prostate cancer is the most common malignancy and the second leading cause of cancer death among men in United States. The American Cancer Society estimates that 180,890 new cases of prostate cancer will be diagnosed and about 26,120 men will die of prostate cancer in 2016, with African-American men having higher incidence and mortality [1]. Prostate cancer is a complex disease that results from genetic and environmental factors, and their interaction. The large variations in prostate cancer rates worldwide and within countries suggest diet may contribute to these variations; however, specific components of the diet have not been well-defined [2, 3].

A dietary factor that has been implicated in prostate cancer pathology is the essential micronutrient zinc. Zinc is widely distributed in the food supply. Major contributors to zinc intake include meats, fish and poultry, dairy, enriched and/or fortified foods, and dietary supplements [4]. The level of zinc in the body is maintained by regulating absorption of exogenous zinc and secretion and excretion of endogenous zinc [5]. In the US, the recommended dietary allowance (RDA) for men who are more than or equal to 19 years old is 11mg/day [6]; however, men consuming vegetarian diets have higher requirements (2 times) since the bioavailability of zinc to the body is reduced with phytate-rich foods (e.g., whole grains, fruits, vegetables). Based on data from the National Health and Nutrition Examination Survey (NHANES 2003–2004 & 2005–2006), most Americans meet the estimated average requirements for zinc [7]. However, non-Hispanic black, men had the lowest median daily dietary zinc intakes compared to non-Hispanic White, and Mexican American in the US population [8].

The levels of zinc in the body are tightly controlled as it is involved in many physiological processes such as enzyme activity, genomic stability, apoptosis, immunity, neurological function, response to oxidative stress, and cell signaling [9]. Normal prostate tissue contains one of the highest concentration of zinc in the body and several studies have shown that malignant prostate tissue lose the ability to accumulate Zinc leading to a decrease in zinc levels compared to normal or hyperplastic tissue (for reviews see [10, 11]). The decrease in prostatic zinc levels correlated with increased Gleason score [12], and level of the zinc uptake transporters (hZIP1, hZIP2 and ZIP3) [13, 14]. In addition, Rishi *et al*. reported decreased expression of hZIP1 and hZIP2 in prostatic tissue of black compared to white males [15]. The role of zinc in prostate cancer pathogenesis has not been elucidated, but there is evidence to suggest that zinc can inhibit energy production, growth and proliferation in normal prostate cells; and suppresses angiogenic and metastatic potentials of malignant prostate cells [11, 16].

Although experimental data supports the protective role of zinc in prostate cancer, epidemiological studies, including case-control, cohort and randomized clinical trials (RCT), have shown mixed results. There are some studies that showed zinc reduces the risk of developing prostate cancer [17–19] and prostate cancer mortality [20]. In addition, an ecologic study from South Carolina found an inverse relationship between soil zinc content and prostate cancer rate [21]. Other studies showed that advanced prostate cancer is associated with high intake of zinc [22–25]. However, many observed that dietary or supplemental zinc intake is not associated with prostate cancer risk or progression [26–34]. Reasons for these conflicting results could be due to different study design, measurements errors for zinc intake, or the lack of an accurate and reliable measurement for zinc status in humans.

The relationship between zinc and prostate cancer is indeed a “critical scientific, medical, and public interest issue” that has yet to be resolved [10]. Few studies assessed the role of zinc intake and prostate cancer among African American men, who are the most affected by prostate cancer [25, 31].

To address this issue, we assessed the association of dietary zinc intake among African Americans from a hospital based case-control study with demographic, lifestyle and clinical characteristics. We also conducted a systematic review of the literature and perform a meta-analysis to summarize the evidence regarding the association between zinc and prostate cancer.

## Materials and Methods

### Case-Control Study

#### Study population

Self-identified African American men aged between 40 and 85 were recruited from the Division of Urology at the Howard University Hospital and Washington D.C. area. Details of the study were previously published [35]. In brief, cases had histological confirmed prostate cancer with a PSA of > 3.5 ng/ml and a positive digital rectal examination (DRE). Controls were healthy, unaffected volunteers with no history of prostate cancer among first-degree relatives from the prostate cancer screening population of the Division of Urology. All participants provided written informed consent. The study and consent forms were approved by the Howard University Institutional Review Board [36, 37]. The present analysis is based on 248 men who provided demographic, lifestyle, dietary, and medical information.

#### Zinc assessment

Usual dietary intake was assessed at the time of recruitment (2000 to 2004) with the use of a block food frequency questionnaire (These self-administered FFQ were adapted from National Health and Nutrition Examination Survey 1999–2001 dietary recall data and validated for use in the African American population to estimate usual zinc intake during the year prior to recruitment into the study [38–40]. The FFQ consisted of 19 food items, 3 supplements questions, and questions to adjust for food fortification practices.

#### Statistical analysis

To compare sample characteristics, t-test for continuous variables and chi-square for categorical variables were used. Unconditional logistic regression was used to calculate the unadjusted and adjusted odds ratio (OR) and 95% confidence intervals (95% CI) associated with prostate cancer and zinc intake. The OR was adjusted for age, PSA, body mass index (BMI), family history of prostate cancer, education, income, fruit, vegetables, iron, calcium intake, smoking and alcohol intake. Zinc intake was analyzed as both continuous and tertiles with cut-points based on its distributions. We used the lowest tertile as the reference group. Linear trends across categories were tested in these models using ordinal variables. The SAS software (SAS Institute Inc., Cary, NC) was used for the analysis.

### Systematic Review and Meta-Analysis

#### Literature search and selection

We conducted a literature search of PubMed, Embase and Cochrane databases from January 1^st^ 1977 to March 31^st^ 2016. The search strategy included a combination of free text and the Medical Subject Headings (MeSH) terms of the following terms: (zinc or ZN) AND (‘prostate cancer’ or ‘prostatic neoplasms’) AND (intake or status or diet or dietary or supplement or supplemental or hair or nail or toenail or plasma or serum or urine). No language restrictions were imposed. Titles and abstracts of identified studies were checked and the full text of relevant studies was assessed for eligibility against the predefined inclusion criteria. We also performed a manual search of references cited in the selected articles and published reviews to search for additional relevant studies and authors were contacted to request missing data. No additional eligible articles were identified in Embase or Cochrane Database of Reviews that we did not identify in PubMed. There was no Cochrane review on the topic. Studies were included in this meta-analysis if they fulfilled the following criteria: 1) presented original data from cohort, case–control, cross-sectional, or RCT studies, 2) the exposure of interest was zinc intake or status, 3) the outcome was prostate cancer occurrence, and 4) the studies reported effect estimates (OR, relative risk (RR), or hazard ratio (HR)) with 95% confidence intervals (95% CI).

#### Data extraction

The following data were extracted from each study: first author’s last name, publication year, study name, study location, ethnic origin of participants, age (mean or range), sample size, zinc intake categories, dietary assessment method, covariates adjusted for in the multivariable analysis, and effect estimates with their 95% CI for each category of zinc. Data extraction was conducted independently by 2 investigators using a standardized data extraction excel sheet, and disagreements resolved by consensus. The reporting protocol for the meta-analysis is shown in S1 Checklist.

#### Statistical analysis

We calculated the standard error for each study using the reported 95% CIs and computed summary RR and 95% CIs from the RR, OR and HR for the highest versus the lowest zinc category using the inverse variance weighted method. Both fixed effects [41] and random effects [42] models were estimated. Chi-square and I^2^ statistics were used to evaluate the statistical heterogeneity among the studies.I^2^ statistic values on the order of 25%, 50%, and 75% are considered as low, moderate, and high, respectively [43].Sources of heterogeneity were assessed by using stratified analyses and meta-regression with source of zinc (dietary, supplemental, total, serum/hair/nail), study design (case-control, cohort, nested case-control and RCT), disease status (early stages (cancer is clinically confined to the prostate gland), late stages (cancer is clinically disseminated outside the prostate gland) [44], mixed/unspecified (all stages combined/not specified by authors), country (USA& Canada, Europe, other), total sample size (<100, 100–500, >500 subjects), publication year (before 1995, 1995–2006, after 2006) and year the recruitment started (before 1984, 1984–1993, after 1993) as potential explanatory factors. We also tested the influence of individual study on the results in sensitivity analyses and performed a cumulative meta-analysis. Cumulative meta-analysis shows the evolution of the estimate of the pooled effect in a meta-analysis with publication year. We examined the publication bias through visual inspection of the Begg’s funnel plots and formally tested using Egger’s regression asymmetry method with results considered to indicate potential small-study bias when P <0.10.

To model the relationship between zinc intake and prostate cancer, we applied a 2-stage random-effects dose-response meta-analysis developed by Orisini et al [42] using generalized least squares for trend (GLST) analysis and restricted cubic splines with three knots at percentiles 10%, 50%, and 90% of the distribution. We computed study-specific RR and 95% CI from the natural logarithms of the effect estimates across categories of zinc intake [45]. The dose–response results are presented for a 100 mg/d increment of zinc intake. The numbers of cases and the denominators in the cohort studies and the median or mean intake for each category were required [42]. Several papers had missing data needed to estimate the dose-response associations. We contacted the authors for the missing information. If we were unable to obtain the data, we derived the missing values using information reported in the papers instead of excluding potentially important studies using recommendations described by Bekkering et al [46]. When the median or mean intake per category was not provided, we assigned the midpoint of the upper and lower boundaries in each category as the average intake. If the lower or upper boundary for the lowest and highest category respectively was not reported, we assigned half the dose to the lower boundary and multiplied the highest level by 1.5. The dose of zinc was not collected by the authors in two studies [18, 25]. For Kristal et al, 1999 [18] we used the dose of zinc from a subsequent paper from the same author (Kristal et al. 2010) [28]; and used the data from Gonzalez et al 2009 [17] for Zhang et al 2009 [25], as the later measured both dose and frequency. All analyses were performed with Stata 11.0 (StataCorp), with a 2-tailed α of 0.05 using a combination of published macros, including metan, metareg, metafunnel, metabias, GLST and MKSPLINE.

## Results

### Case-Control Study

Table 1 summarizes the descriptive characteristics of the study participants in the case-control study. The cases had lower educational level and income and less likely to eat red meat and vegetables or be current drinker. The daily zinc intake was lower in the case than control (p = 0.06). However, prostate cancer cases did not differ significantly from the healthy controls for BMI, family history of prostate cancer, daily dietary intake of total iron, total calcium, total fat and mean energy intake. We examined the association between total zinc intake and prostate cancer as a continuous variable or divided into tertiles (<6.5, 6.5–12.5, >12.5mg/day). In Table 2, we report crude and adjusted prostate risk estimates in relation to total zinc intake. We did not find an association between zinc intake (treated as continuous variable) and prostate cancer after adjusting for age, food energy, body mass index, education, income, smoking history, alcohol, total fat, family history of prostate cancer, PSA levels, vegetables and fruit servings per day, total calcium and iron levels (OR = 0.97, 95% CI: 0.46, 2.1). When we examined tertiles of zinc intake, we observed a non-linear increase in the odds of prostate cancer with increasing zinc intake (p-trend of adjusted model = 0.6).

**Table 1:** Demographic and health-related characteristics of the Washington D.C. prostate cancer study by outcome.

	Cases (n = 127)	Controls (81)	P-Value
**Mean (SD) Age at diagnosis, years**	66.5 (9)	66 (10.7)	0.75
**Income, %**			
$0- $29,999	57	44	0.09
$30,000-$59,999	26	28	
over $60,000	17	28	
**Education, %**			
less than high school	63	46	0.01*
High school or more	37	54	
**Body Mass Index at diagnosis (kg/m**^**2**^**), %**			
Normal weight (<25)	33	29	0.8
Over weight (25–30)	39	42	
Obese (>30)	28	29	
**Smoking status (%)**			
Current smoker	20	25	0.6
Former smoker	47	40	
Never smoker	33	35	
**Alcohol use (%)**			
Current Drinkers	37	52	0.08
Former Drinker	35	23	
Non-drinkers	28	25	
**Family history of Prostate cancer (%)**			
Yes	19	14	0.42
**PSA ng/mL at the evaluation or diagnosis (%)**			
0–2.5	19	51	<0.0001*
2.6–9.9	44	43	
10–19.9	15	2	
>20	22	4	
**Zinc, Daily intake (±SD)**			
Total Zinc (mg)	10.8 (7.4)	14.3 (15.4)	0.06
Zinc from supplements (mg)	5.2 (11.9)	6.7 (12.5)	0.4
**Potential modifiers of zinc intake, Daily intake (±SD)**			
Red meat (servings)	2.1 (1.4)	3.1 (2.9)	0.0008*
Vegetables (servings)	3.3 (2.3)	4.2 (3.5)	0.03*
Fruits (servings)	1.8 (1.2)	1.8 (1.4)	0.8
Total Iron (mg)	14.9 (9.2)	17.2 (14.4)	0.19
Total Calcium (mg)	730.6 (470.1)	736.8 (611.8)	0.94
Energy intake (kcal)	2029.2 (1225.7)	2387.9 (1890.1)	0.13
Dietary total fat (g)	81.6 (56.3)	96.8 (80.5)	0.14
Saturated fat	23.1 (16.2)	26.4 (23.4)	0.26
Polyunsaturated fat	21.2 (15.51)	25.9 (20.8)	0.08
Monounsaturated fat	30.9 (22.3)	36.6 (31.7)	0.16

* p<0.05

**Table 2:** Crude and adjusted prostate cancer estimates.

Total zinc intake	Unadjusted OR (95% CI)	Adjusted OR (95%CI)^a^
**≤6.50 (mg/day)**	Reference	Reference
**6.5–12.50**	1.51 (0.57, 2.3)	1.8 (0.6, 5.6)
**>12.5**	0.81 (0.41, 1.61)	1.3 (0.2, 6.5)
	^b^p-trend = 0.5	p-trend = 0.6
**Continuous (10mg/day)**	0.75 (0.57, 0.99)	0.97 (0.46, 2.1)

a) OR generated from logistic regression model adjusting for age, food energy, meat consumption. body mass index, education, income, smoking history, alcohol, total fat, family history of prostate cancer, PSA levels, vegetables and fruit servings per day, total calcium and iron levels.

b) p value for trend

### Meta-Analysis

**Fig 1:**
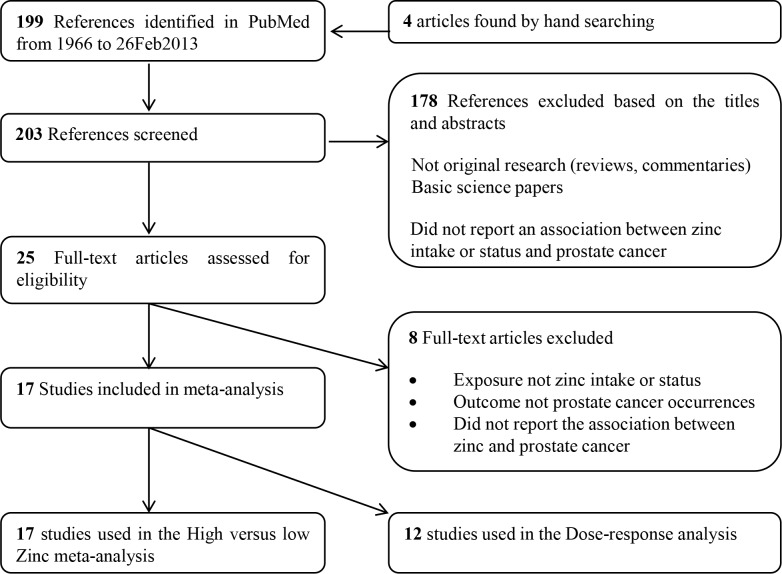
Flow diagram of systematic literature search on zinc and the risk of prostate cancer.

We also conducted a systematic review of the literature and performed a meta-analysis to summarize and pool the findings from multiple studies that measured the association between zinc and prostate cancer risk in the general population. We identified a total of 199 articles from our systematic review and 4 from hand-searching. We scanned the titles and abstract of the 203 articles to remove commentaries, reviews, basic science papers resulting in 25 articles. The full text-articles of the 25 articles that assessed the association between zinc and prostate cancer were reviewed. Eight articles were excluded as they did not report the association between zinc and risk of prostate cancer [17, 20, 21, 47–52] and we included the remaining 17 articles in our meta-analysis [17–19, 22–34, 53] (Fig 1).

### Characteristics of the Included Studies

The studies that met our inclusion criteria included 3 cohort, 2 nested case-control, 11 case-control, and 1 randomized clinical trial with a total of 111,199 participants and 11,689 cases of prostate cancer, were used for the meta-analysis. Details of each of these studies are summarized in Table 3. The numbers of participants in each study ranged from 143 to 46,974. The studies were generally from the United States (n = 10) or European countries (n = 4); 2 studies was based in China and 1 from Malaysia. Four studies assessed zinc status from nail, hair and plasma, and the rest used FFQ to estimate zinc intake. Dose of zinc in most of the studies was mg/day. All of the studies adjusted for age, as age is an established risk factor for prostate cancer.

**Table 3:** Characteristics of papers included in the meta-analysis.

First Author, year	Study design	Population (Study period)	Cases #	Total #	Age (years) range/mean	Source of Zinc	Highest category	Stratification/Adjustment variables
**Kolonel, 1988**	case-control	Hawaii, USA: 37% Japanese, 31% white (1977–1983)	452	1351	>70 (43%) & ≤70 (57%)	Dietary & supplements	>100 (mg/day)	Stratified by Age (<70 & ≥70); zinc intake (dietary & total); Adjusted for ethnicity
**West, 1991**	case-control	Utah, USA: LDS members (1984–1985)	358	1037	45–74	Dietary	>16 (mg/day)	Matched by age and residence; stratified by age (45–67; 68–74) and pathology (all tumors, aggressive tumor; adjustment for energy and age
**Andersson, 1996**	case-control	Sweden (1989–1994)	526	1062	45–74	Dietary	>13.5 (mg/day)	Stratified by pathology (all stages & advanced); adjusted for age, energy
**Key, 1997**	case-control	UK (1990–1994)	328	656	68.1	Dietary	≥ 11 (mg/day)	Matched for age; adjusted for social class
**Vlajinac, 1997**	case-control	Serbia (1990–1994)	101	303	70.5 cases & 71.5 control	Dietary	NR	Matched for age, hospital admittance and place of residence; adjusted for energy, protein, total fat, saturated fatty acids, carbohydrates, total sugar, fiber, retinol equivalent, alpha-tocopherol, folic acid, vitamin B12, potassium, calcium, phosphorous, magnesium and iron
**Lee, 1998**	case-control	China (1989–1992)	133	398	50–80	Dietary	NR	Adjusted for region, fat, carotenoids and selenium
**Kristal,1999**	case-control	Washington, USA: 95–98% white (1993–1996)	697	1363	40–64	Supplements	≥ 7 (frequency/week)	Stratified by stage and grade; adjusted for age, energy, fat, race, family history, BMI, PSA testing, education
**Platz, 2002**	nested case-control	CLUEII cohort, USA (1989–1996)	115	342	59–74	Toe nail	259.1 (ppm)	Matched on age, race, date of blood collection and size of toenail clipping; adjusted for education, adult height, current BMI, BMI at age 21, father or brother with prostate cancer, cigarette smoking, and multivitamin use
**Litzmann, 2003**	cohort	Health professionals cohort, USA (1986–2000)	2901	46974	44–66	Supplements	101 mg	Stratified by stage; adjusted for age, energy, BMI, height, smoking, family history, physical activity, aspirin use, dietary calcium, supplemental calcium, fructose, supplemental vitamin E, tomato-based foods, fish, red meat, and alpha -linolenic acid.
**Meyer, 2005**	RCT	SU.VI.MAX trial, Canada (1994–2004)	101	4830	45–60	Serum	≥ 13.4 mmol/L	Matched for age, PSA, smoking, BMI, serumβ-carotene, α-tocopherol, vitamin C, Selenium
**Gallus, 2007**	case-control	multicenter hospital study from Italy (1991–2002)	1294	2745	46–74	Dietary	>15.65 (mg/day)	Adjusted for age, study center, education, physical activity, family history, BMI and total energy intake
**Zhang, 2009**	case-control	Case-Control Surveillance Study: 77–84% White, USA (1976–2006)	1706	4110	40–79	Supplements	≥10 years	Adjusted for matching variables age, study center, year of interview and race, and for education, BMI, alcohol, current smoking, family history, use of other vitamins & mineral supplements.
**Gonzalez, 2009**	cohort	Vitamin and Lifestyle cohort: 93–94% white, USA (2000–2004)	832	35244	50–76	Dietary & supplements	152 mg/day	Stratified by zinc category, stage, grade &vegetable and fruit intake; adjusted for education, race, family history, PSA-test within the 2 years prior to baseline, & current multivitamin use.
**Kristal, 2010**	cohort	Prostate cancer prevention trial: 93–94% white, USA& Canada (1994–2003)	1703	9559	cases: 63.6 & controls: 62.6	Dietary & supplements	>22 mg/day	Adjusted for age, race, family history, treatment arm, BMI and pathology
**Karimi, 2012**	case-control	Hospital-based study from Malaysia: 47% Malays, 33% Chinese and 20% Indians (2010–2012)	50	50	50–86	Hair and Nails	hair: >3.75 mg/g & Nails: >3.32 mg/g	Matched for age and ethnicity
**Park, 2013**	Nested case-control	Multiethnic cohort study from Hawaii and California USA: 46% AA, 20% Japanese (1993–2006)	392	1175	45–75	Serum	>102.5 μg/dl	Matched for geographic location, race, birth year, date of blood draw, time of blood draw, and fasting hours prior to blood draw, family history, BMI, and education

Abbreviations: AA: African American, RCT: Randomized control-trial, BMI: body mass index

### Highest versus Lowest Zinc Category Meta-Analysis

**Fig 2:**
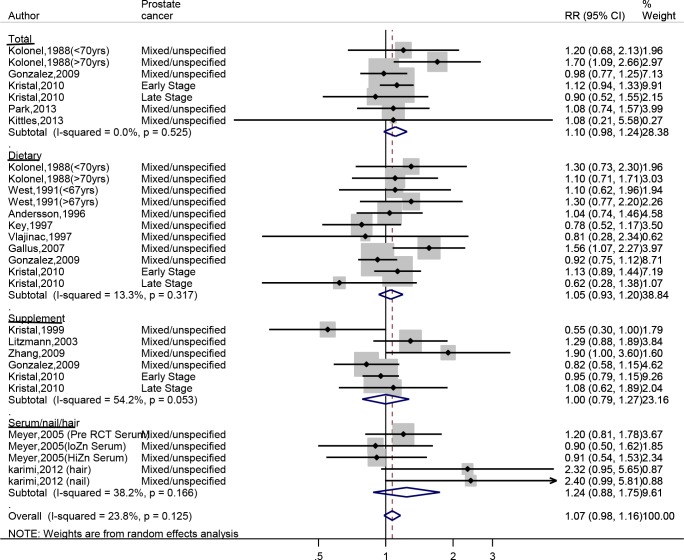
Forest plot of included studies for the highest versus lowest meta-analysis, stratified by zinc intake (dietary, supplement, and total) or zinc status (serum, nail, and hair).

**Fig 3:**
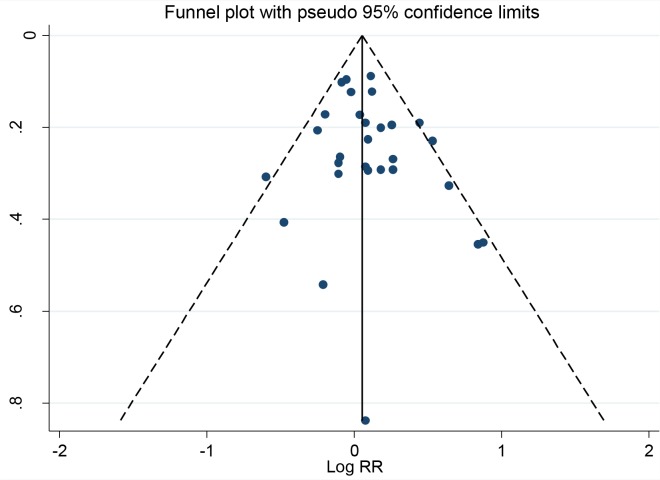
Funnel plot of studies examining the association between zinc and prostate cancer incidence as a test for publication bias.

**Fig 4:**
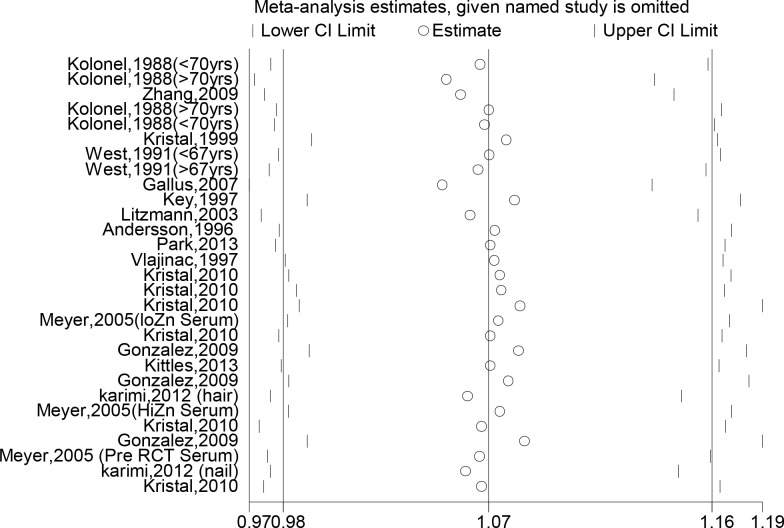
Sensitivity analysis investigating the influence of each individual study on the overall meta-analysis of zinc and risk of prostate cancer. The meta-analysis of all studies except the “omitted” study named on the left margin is presented as a horizontal confidence interval. The full, “combined” results are shown as the solid vertical lines.

We performed a meta-analysis including our case-control study comparing the highest to the lowest zinc intake category (Fig 2). The random effect summary risk estimates from the 17 studies indicated that the combined high zinc consumption increased the risk of prostate cancer by 7% (OR _high vs. low_ = 1.07, 95% CI: 0.98, 1.64). Visual inspection of the funnel plot did not identify any substantial asymmetry (Fig 3) and the Egger linear regression test also indicated no evidence of significant publication or small-study bias (p = 0.679). Sensitivity analysis showed that no specific study has a strong influence on the summary estimate as the overall OR after exclusion of any individual study was between 0.97 and 1.19 (Fig 4).

Subgroup analyses were conducted to further examine the relationship between zinc and prostate cancer (Table 4). There was a 10% increase in the risk of prostate cancer when total zinc intake was examined (RR = 1.10, 95% CI: 0.98, 1.24) that was borderline statistically significant (p = 0.107) and low heterogeneity (I^2^ = 0%) among the all studies. The increased risk was absent for the relationship between supplemental zinc intake and prostate cancer (RR = 1, 95% CI: 0.79, 1.27) with strong evidence for heterogeneity among these studies (I^2^ = 54.4). We observed greater extent of homogeneity among studies assessing dietary (I^2^ = 22%) or total zinc intake (I^2^ = 0%) compared to supplemental intake (I^2^ = 54%) or zinc status (I^2^ = 38%). There was 21% increase in the risk of prostate cancer among case-control studies compared with cohort studies (p = 0.04) with medium heterogeneity (I^2^ = 38%). The source of this heterogeneity seems to be the studies where supplemental zinc was assessed.

**Table 4:** Summary of the meta-analyses for the association of zinc and prostate cancer.

	n	RR	(95%CI)	p-value	I^2^	p-value*
**Highest versus lowest meta-analysis**						
**All studies**	29	1.07	(0.98,1.64)	0.127	23.8%	0.125
**By Zinc category**						
**Zinc intake (dietary, supplemental, total)**	24	1.05	(0.97,1.15)	0.232	21.6%	0.169
Dietary	11	1.05	(0.93,1.20)	0.416	13.3%	0.317
Supplemental	6	1.00	(0.79,1.27)	0.999	54.4%	0.053
Total	7	1.10	(0.98,1.245)	0.107	0%	0.525
**Zinc status (serum/toes /hair)**	5	1.24	(0.88,1.75)	0.166	38.2%	0.166
**By Pathology**						
Early	3	1.06	(0.95,1.12)	0.329	0%	0.374
Late	3	0.90	(0.63,1.28)	0.553	0%	0.536
Mixed/unspecified	23	1.10	(0.98,1.23)	0.113	32.7%	0.067
**By Study design**						
Cohort and nested case-control	14	1.01	(0.94,1.10)	0.735	0%	0.745
Case-Control	15	1.21	(1.01,1.46)	0.041	39%	0.059
**Total Zinc only**						
**By Pathology**						
Early	1	1.12	(0.94,1.33)	0.201	-	-
Late	1	0.90	(0.52,1.55)	0.704	-	-
Mixed/unspecified	5	1.13	(0.93,1.38)	0.228	12.5%	0.334
**By Study design**						
Cohort and nested case-control	4	1.06	(0.93,1.21)	0.364	0%	0.765
Case-Control	3	1.47	(1.04,2.07)	0.029	0%	0.6
**Dietary Zinc only**						
**By Pathology**						
Early	1	1.13	(0.89,1.44)	0.32	-	-
Late	1	0.62	(0.28,1.38)	0.24	-	-
Mixed/unspecified	9	1.06	(0.91,1.22)	0.47	15.5%	0.304
**By Study design**						
Cohort and nested case-control	3	0.98	(0.80,1.21)	0.841	33.1%	0.224
Case-Control	8	1.123	(0.95,1.33)	0.173	2.7%	0.409
**Supplementary Zinc only**						
**By Pathology**						
Early	1	0.95	(0.79,1.15)	0.592	-	-
Late	1	1.08	(0.62,1.90)	0.788	-	-
Mixed/unspecified	4	1.02	(0.65,1.58)	0.944	71.90%	0.014
**By Study design**						
Cohort and nested case-control	4	0.98	(0.84,1.15)	0.788	8.30%	0.352
Case-Control	2	1.02	(0.30,3.43)	0.978	86.90%	0.006

n = number of observations; RR = relative risk; CI = confidence interval; I^2^ = Heterogeneity,

* = p-value for heterogeneity

### Dose-Response Meta-Analysis

**Fig 5:**
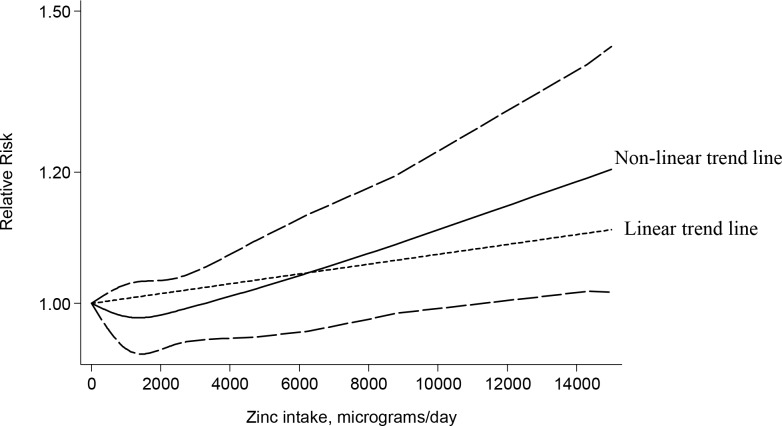
Dose-response relations between zinc intake and RR of prostate cancer (P for nonlinearity = 0.0022). The fitted nonlinear trend is represented by the solid line with the 95% confidence intervals line in long dashes. Lines with short dashes represent the linear trend.

Twelve studies were included in the dose–response analysis [17, 18, 22–28, 33, 34]. Publication bias was not evident with Egger’s test (p = 0.84). The result of the dose-response meta-analysis indicates that there is no association between zinc intake and prostate cancer (RR _100mg/day increase_ = 1.07, 95% CI: 0.90, 1.28). If we assume that the relationship between zinc and prostate cancer is linear, an intake of 100 mg/day of zinc would increase the risk of prostate cancer by 7%. However, the relationship between zinc intake and prostate cancer is not linear (non-linearity test p = 0.0022) (Fig 5).

## Discussion

Prostate cancer disproportionately affects men of African descent. Protective role of zinc in prostate cancer has been observed in animal and in vitro studies but epidemiological studies, including case-control, cohort and RCT, have shown mixed results. To address this issue, we assessed the association of dietary zinc intake among African Americans from a hospital based case-control study with demographic, lifestyle and clinical characteristics. We also conducted a systematic review of the literature and perform a meta-analysis to summarize the evidence regarding the association between zinc and prostate cancer.

The results of our case-control study suggest that there is a non-linear increase in risk of prostate cancer with increasing intake of zinc which is not statistically significant. Although this is the first study to examine the relationship between zinc intake and prostate cancer in African American, this is not an unusual finding as an increase in risk of prostate cancer has been previously reported in response to other vitamins and minerals [22–25]. For instance, vitamin E supplementation was shown to be associated with increased prostate cancer among the participants of the Selenium and Vitamin E Cancer Prevention Trial (SELECT) [54] depending on the basal status or interactions with other nutrients [55]. Kolenko et al [56] in a recent report explains the increased risk of prostate cancer with increasing intake of zinc as a result of the mechanisms regulating zinc hemostasis and bioavailability.

We conducted a meta-analysis to systematically summarize the results from multiple studies to generate a pooled estimate of the effect of zinc and prostate cancer among the general population. We performed an overall and dose-response meta-analyses to examine the shape of the relationship between zinc intake and risk of prostate cancer. The high versus low meta-analysis showed a statistically non-significant positive association between zinc intake and prostate cancer (summary estimate OR = 1.07, 95% CI: 0.98, 1.16). Subgroup analysis showed when looking at dietary (RR = 1.05 (0.93, 1.2)), supplemental (RR = 1.0 (0.79, 1.27)) or total zinc (RR = 1.10 (0.98, 1.245), there was no apparent association with prostate cancer risk. However, there was a 24% increase risk of prostate cancer associated with increased zinc intake measured in serum [30] and hair/nail [53]. The observed increased association between levels of zinc status (serum and nail) and prostate cancer could be a result of a better estimation of zinc exposure, eliminating recall bias. On the other hand, it could be due to the lack of correlation between zinc biomarkers and zinc status [57].

Other limitations of our study should also be acknowledged. Our case-control study has small sample size and the recall bias associated with case-control studies and FFQ assessments. In the meta-analyses errors in measurement of zinc intake could have attenuated individual study results and led to the null association between zinc intake and risk of prostate cancer. All the studies in our analysis (except for two) assessed zinc intake using FFQ, several of which have been validated with reasonable reproducibility and validity [38–40]. Misreporting of intake was still inevitable. Although we did not observe publication bias, it could still be present as the published results might not be representative of the conducted studies. The presence of high heterogeneity among the studies that assessed supplemental zinc is very evident (I^2^>71%) but using random effect models and performing sensitivity analysis showed that this high level of heterogeneity among the studies did not influence the summary estimate.

In summary, we found that low dietary intake of zinc in our case-control study among African Americans showed an increase in prostate cancer risk albeit statistically non-significant. However, the pooled estimates from published studies indicated that zinc intake was not associated with prostate cancer risk. This inconsistency could be explained by the study limitations we mentioned above including small sample size and the recall bias however, it is likely that the major explanation resides within the population heterogeneity in the studies we included in the meta-analysis. There is a lack in studies that examined the association between zinc intake and prostate cancer exclusively in African American. All the studies we included in the meta-analysis examined zinc intake in the general population with no racial/ethnic stratification. An important factor that has not been considered in these epidemiological studies is the heterogeneity of zinc uptake by the prostate gland among different populations. This is supported by previous studies reporting a significant downregulation of the two major zinc transporters, hZIP1 and hZIP2, in normal prostate tissues from African American men when compared with age-matched white men [15]. The reason behind this phenotype is thought to be evolutionary. Since Africa is a mineral-rich continent, Africans may have genetically downregulated their zinc absorption capacity to avoid high toxic levels of zinc that might result in various serious neurodegenerative and biochemical disorders [58]. It has been also shown that the expression of zinc transporters correlates significantly with zinc levels in prostate gland tissues [59]. These findings indicate that African American men need to have higher zinc intake than their White counterparts and might also explain the association we encountered between low zinc intake and prostate cancer in African American while no association was found in the meta-analysis that comprised heterogeneous populations. Thus, the current study indicates the imminent need to conduct further studies that represent racial/ethnic minorities, especially African American who are more susceptible to have zinc deficiency and unintentionally underrepresented in the current literature, to examine the association between zinc intake and the risk of prostate cancer. Also, large epidemiologic studies based on prospective zinc data, preferably using biologic samples (eg, toenails) with repeated collection over time to better reflect long-term exposures, could give a better insight into this critical question.

## Supporting Information

S1 PRISMA Checklist (DOCX)
